# Facile UV-Induced Surface Covalent Modification to Fabricate Durable Superhydrophobic Fabric for Efficient Oil–Water Separation

**DOI:** 10.3390/polym15112505

**Published:** 2023-05-29

**Authors:** Mengmeng Zhou, Xiaohui Liu, Fengjiao Xu, Yongbing Pei, Lianbin Wu, Long-Cheng Tang

**Affiliations:** Key Laboratory of Organosilicon Chemistry and Material Technology of Ministry of Education, College of Material, Chemistry and Chemical Engineering, Hangzhou Normal University, Hangzhou 311121, China

**Keywords:** durable, superhydrophobic, oil–water separation, fabric

## Abstract

In this work, a durable superhydrophobic fabric was fabricated by using a facile UV-induced surface covalent modification strategy. 2-isocyanatoethylmethacrylate (IEM) containing isocyanate groups can react with the pre-treated hydroxylated fabric, producing IEM molecules covalently grafted onto the fabric’s surface, and the double bonds of IEM and dodecafluoroheptyl methacrylate (DFMA) underwent a photo-initiated coupling reaction under UV light radiation, resulting in the DFMA molecules further grafting onto the fabric’s surface. The Fourier transform infrared, X-ray photoelectron spectroscopy and scanning electron microscopy results revealed that both IEM and DFMA were covalently grafted onto the fabric’s surface. The formed rough structure and grafted low-surface-energy substance contributed to the excellent superhydrophobicity (water contact angle of ~162°) of the resultant modified fabric. Notably, such a superhydrophobic fabric can be used for efficient oil–water separation, for example a high separation efficiency of over 98%. More importantly, the modified fabric exhibited excellent durable superhydrophobicity in harsh conditions such as immersion in organic solvents for 72 h, an acidic or alkali solution (pH = 1–12) for 48 h, undergoing laundry washing for 3 h, exposure to extreme temperatures (from −196° to 120°), as well as damage such as 100 cycles of tape-peeling and a 100-cycle abrasion test; the water contact angle only slightly decreased from ~162° to 155°. This was attributed to the IEM and DFMA molecules grated onto the fabric through stable covalent interactions, which could be accomplished using the facile strategy, where the alcoholysis of isocyanate and the grafting of DFMA via click coupling chemistry were integrated into one-step. Therefore, this work provides a facile one-step surface modification strategy for preparing durable superhydrophobic fabric, which is promising for efficient oil–water separation.

## 1. Introduction

In recent decades, the high demand for petroleum products has imposed a huge environmental burden. The oily waste water has seriously affected the marine environment and ecosystem, owing to frequent oil spills, oil transportation, and oil refineries [1]. A relatively recent oil spill happened in the Gulf of Mexico in 2010, which was documented as the biggest accidental ocean leak in U.S. history, severely affecting marine life and coastal wetlands [2]. In addition to the oil spill, the leakage of industrial oily wastewater and water-insoluble organic solvents (such as benzene, toluene, and dichloromethane) also significantly impended public health and security [3], which impels the development of approaches and materials for separating oil–water mixtures as a greatly concerned issue [4,5]. Herein, aiming at the separation and collection of oil from oily water, developing novel approaches and materials to achieve efficient oil–water separation has become one of the hottest research topics [6,7,8,9].

To date, diverse techniques and materials have been employed for recovering the oily waste water. The traditional methods include gravity separation [10], air flotation [11], in situ burning [12], bioremediation [13], adsorption [14], etc. However, not only massive time, materials, and other resources are engaged in these traditional cleanup methods, but also high costs, complex processes [15], and low separation efficiency [16]. Since Jiang et al., first reported coating a superhydrophobic/superoleophilic film on mesh to recover oily water due to its special wettability, which can selectively absorb oil while repelling water [17], superhydrophobic materials have gained considerable attention, thereby a variety of superhydrophobic/superoleophilic materials have been developed, including porous foam [18], sponges [19,20], fabrics [21,22,23,24], aerogels [25], and membranes [26,27,28,29]. In particular, the cellulose-based fabrics such as wovens and cotton show great prospects because of their superiorities such as being light-weight and low-cost, their excellent recyclability, and their mechanical strength.

Generally, a superhydrophobic surface refers to the condition where a water droplet can stand on the material’s surface with a water contact angle (WCA) greater than 150° [30]. This superhydrophobic surface is generally derived by increasing the surface roughness and decreasing the surface tension [31,32]. As is well known, fabric is naturally hydrophilic; thus, surface modification is commonly required to become superhydrophobic prior to finding application in oil–water separation. Various strategies including dip-coating [33], chemical vapor deposition [34], and plasma treatment [35] have been utilized to fabricate superhydrophobic fabric. Depositing or anchoring nanoparticles onto the fabric’s surface is involved in these fabrication methods. For example, Dilshad et al., anchored TiO_2_ nanoparticles onto the fabric’s surface using aminopropyltriethoxysilane, then treated the fabric with myristic acid [24]; Yan et al., immersed the pre-cleaned textile fabric into a Fe_3_O_4_/TiO_2_ solution [36]; Zhou et al., dip-coated fabric samples with the coating solution of SiO_2_ nanoparticles and poly-dimethylsiloxane [37]; then, superhydrophobic fabrics were obtained after drying. Typically, these superhydrophobic fabrics require recycling and inevitably require laundry washing. Unfortunately, these superhydrophobic surfaces are vulnerable to losing their superhydrophobicity under extreme environments, mechanical wear, chemical corrosion, etc. To enhance the superhydrophobic durability, constructing covalent bonds between the fabric and nanoparticles or low-surface-energy substances is commonly used [38,39,40]. For instance, Hou et al., immersed the fabric’s surface into the solution of mercapto silanes for 2 h to make the thiol group graft onto the fabric’s surface via a covalent bond, then grafted ene-containing polyhedral oligomeric silsesquioxane (POSS) nanoparticles onto the pre-treated cotton fabric via the thiol-ene click reaction; the constructed rough structure and grafted low-surface-energy substance made the fabric become superhydrophobic [41]. Fang et al., used a similar method to obtain mercapto-silane-pre-treated fabric, then grafted vinyl-capped SiO_2_@Fe_3_O_4_ nanoparticles onto the fabric’s surface [40]. In this way, the established covalent bond interaction among the fabric and nanoparticles or low-surface-energy substances thereby could play the role of enhancing the fabric’s superhydrophobic durability against mechanical damage, laundry washing, etc. However, these methods suffer from complex steps, including a long duration of the mercapto silane treatment in advance and the necessary component of the nanoparticles such as POSS or SiO_2_, which would bring negative effects such as increasing the fabric’s weight, blocking the fabric’s porous structure, then decreasing the oil–water separation efficiency and impacting the feel of the fabric.

In this work, we present a facile, one-step, highly efficient surface modification strategy to prepare superhydrophobic fabric with excellent durability, in which 2-isocyanatoethylmethacrylate (IEM) and dodecafluoroheptyl methacrylate (DFMA) were grafted onto the fabric’s surface via click chemistry under the aid of UV light. During the reaction process, the anchoring of IEM molecules onto the fabric’s surface through the alcoholysis reaction between the isocyanate and hydroxyl groups, together with the covalent grafting of DFMA via the click reaction, were integrated into one step under the aid of UV light. The anchored IEM molecules can serve as the “bridge” to graft fuDFMA molecules onto the fabric’s surface. The grafted IEM and DFMA molecules formed a rough structure, together with the low-surface-energy fluoride materials; thus, the fabric became superhydrophobic. The superhydrophobic fabric was used for oil–water separation, and the separation efficiency for various oil–water mixtures was tested to evaluate its separation performance. Furthermore, the durability of the prepared superhydrophobic fabric against external damage such as laundry washing, tape-peeling, and abrasion was also investigated.

## 2. Experimental Section

### 2.1. Materials

Commercial cellulose-based fabric (warp density: 45 threads per cm; weft density: 21 threads per cm, with 153 g/m^2^) was supplied by Transfar Co., Ltd. (Hangzhou, China). 2-isocyanatoethylmethacrylate (IEM) in analytical grade was obtained from Meryer company (Shanghai, China). Dodecafluoroheptyl methacrylate (DFMA) of analytical grade was provided by Jiuding Chemical Technology Co., Ltd. (Shanghai, China). 2-hydroxy-2-methylpropiophenone (HMPF) was bought from Energy Chemical Co., Ltd. (Shanghai, China). Dichloromethane (DCM), ethyl acetate, anhydrous ethanol, *n*-heptane, acetone, carbon tetrachloride, gasoline (95#), ethyl acetate, and toluene were supplied by Sinopharm Chemical Reagent Co., Ltd. (Shanghai, China). UV light equipment was supplied from Haite Naide Co. Ltd. (UV-2LD, Shenzhen, China). All reagents were analytical grade and directly used without further purification.

### 2.2. Fabrication of the Superhydrophobic F-IEM@fabric

The preparation formula of F-IEM@fabric is shown in Table 1. Typically, the F-IEM@fabric was prepared as follows. First, the commercial fabric (4 cm × 4 cm × 1 mm) was repeatedly cleaned in de-ionized water several times to remove impurities on the fabric’s surface. After the pre-cleaned fabric was ultrasonically treated in an absolute ethanol solution for 30 min to obtain a hydroxylated fabric (designated as HO-Fabric), the HO-Fabric was completely immersed into the well-mixed solution composed of 0.02 mol IEM, 0.02 mol DFMA, 0.2 g photo-initiator HMPF, and 40 g DCM. Accompanied by magnetic stirring, the mixture was exposed to ultraviolet light of a wavelength of 365 nm at an intensity of 40 W with a distance of 30 cm for 30 min. Afterwards, the fabric was taken out and rinsed with DCM to remove residual reactants, and placed in an oven at 60 °C for 3 h to dry thoroughly. The resultant target fabric was designated as F-IEM@fabric.

### 2.3. Characterization

The Fourier transform infrared (FTIR) spectrum was recorded on a spectroscope (Antaris, Nicolet 7000, Thermo Fisher, Waltham, MA, USA) in the range of 4000 to 400 cm^−1^. The surface morphology of the fabric before and after modification were comprehensively observed by using a Zeiss ultra plus field emission scanning electron microscope (FE-SEM, Zeiss, sigma300, Oberkochen, Germany). The surface chemical elements and content were determined by using energy dispersive X-ray spectroscopy (EDS) and SCIENTIFIC ESCALAB 250Xi High-resolution X-ray photoelectron spectra (XPS) measurements (Thermo Fisher, Waltham, MA, USA). The water contact angle (WCA) of the fabric was measured by using the DSA30 video optical contact angle analyzer (KRUSS, Hamburg, Germany), and the results are reported as the average value from three different sites on the fabric’s surface.

### 2.4. Oil–Water Separation Efficiency

A simple set of equipment was set up to test the oil–water separation efficiency of the F-IEM@fabric. Two round glass tubes with openings at both ends were used, which had a diameter of about 3 cm and a length of about 10 cm. The superhydrophobic F-IEM@fabric was sandwiched between these two glass tubes and fixed on an iron frame using a clip. The F-IEM@fabric was used to separate the mixture solution of 100 mL of water and 100 mL of solvent. The solvent–water mixture was poured slowly from the top into the glass tube, and the solvent permeated into the fabric while the water was repelled. When the separation was complete, the separated solvents were collected. The separation efficiency (*E*) was calculated to evaluate the oil–water separation performance of the target fabric by the formula as follows:

*E* = (*V*/*V*_0_) × 100%

where *V* and *V*_0_ represents the volume of the collected solvents from the mixture after separation, and the initial volume of solvent before separation, respectively.

### 2.5. Durability of the Superhydrophobic F-IEM@fabric

For the durability in harsh conditions such as chemical solvents and strong acidic/alkali solutions, the F-IEM@fabric was immersed into various organic solvents and aqueous solutions with a pH ranging from pH = 1 to pH = 12 for 48 h. Then, the fabric was taken out to dry in an oven, then the WCA was measured to evaluate the chemical durability.

For the durability against UV irradiation and high-/ultra-low temperature, the F-IEM@fabric was exposed to UV light with a wavelength of 365 nm and placed at the different temperature conditions for 30 min, respectively. The F-IEM@fabric was taken out and its WCA measured.

For the durability against mechanical damage, a tape-peeling-off test was conducted for the F-IEM@fabric. First, the F-IEM@fabric was fixed on a glass plate by double-sided tape. In order to achieve a complete contact between the fabric and tape, an external force of 20 kPa was exerted onto the tape, then the F-IEM@fabric was peeled from the tape after staying for 1 min; the whole process was defined as one cycle. The above operation was repeated for 100 cycles, and the WCA was measured each 10 cycles.

For the durability against abrasion, the F-IEM@fabric was placed on sandpaper with a 300 mesh, then a 100 g balancing weight was exerted on the fabric. The F-IEM@fabric was moved forward 20 cm at a rate of 20 cm/min under external drag force, which was defined as one cycle. A total of 100 cycles were operated, and the WCA was measured each 10 cycles.

## 3. Results and Discussion

### 3.1. Fabrication of Superhydrophobic F-IEM@fabric

**Figure 1:**
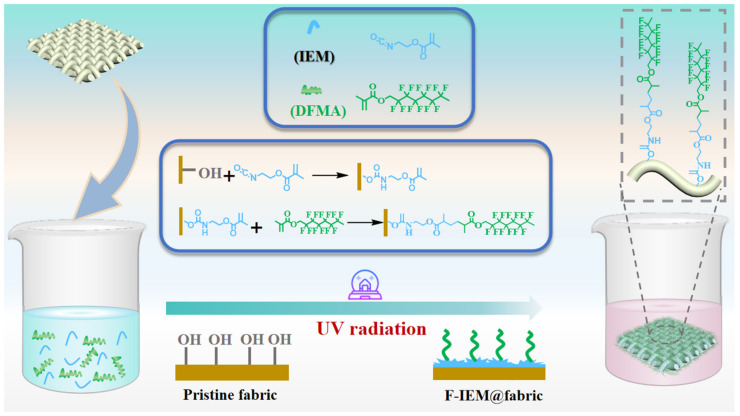
Schematic diagram of preparation of superhydrophobic F-IEM@fabric.

In this work, a superhydrophobic fabric was developed via a facile one-step surface modification strategy, which is illustrated in Figure 1. Serving as the starting material, the pristine cellulose fabric was ultrasonically treated in an absolute ethanol solution in advance; the hydroxylation effect would endow the fabric’s surface with a large amount of hydroxyl groups (–OH), then the HO-Fabric was transferred to a well-mixed solution of IEM, DFMA, and the photo-initiator HMPF. The highly reactive isocyanate (–NCO) groups in IEM reacted with the –OH groups to form the urethane group (NH–CO–O), resulting in the IEM anchoring onto the surface of the fabric. Simultaneously, the double bonds in IEM and DFMA underwent a photo-initiated radical coupling reaction under the assistance of UV light radiation, resulting in the DFMA molecules further grafting onto the fabric’s surface. As a result, the hydrophobic fluorine-containing compounds gathered on the fabric’s surface. Upon the completion of the reaction, the target fabric was obtained after washing with DCM to remove the residuals followed by drying, designated as the F-IEM@fabric for convenience.

**Figure 2:**
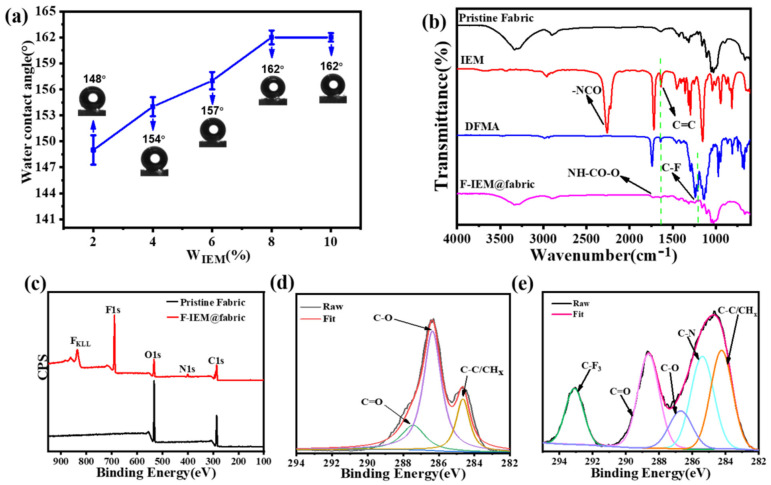
(**a**) The WCA of F-IEM@fabric at various IEM mass concentrations. (**b**) FTIR spectra of pristine fabric, IEM, DFMA, and F-IEM@fabric. (**c**) XPS spectra of pristine and F-IEM@fabric. High-resolution C1s spectra of (**d**) pristine fabric and (**e**) F-IEM@fabric.

During the fabrication process, the coupling reaction happened between the C=C bonds in the IEM and DFMA molecules, respectively. The concentrations of IEM and DFMA were the crucial factors affecting the surface property of the F-IEM@fabric. It is essential to investigate these key parameters to obtain the best characteristics. Considering that the expected grafting substances on the fabric’s surface were derived from IEM and DFMA through radical coupling reactions, the theoretical and suitable molar ratio of IEM and DFMA could be set as 1:1. To further investigate the effect of the IEM and DFMA concentration on the surface property of the F-IEM@fabric, the mass concentration of IEM was utilized for convenience. The WCA of the resultant F-IEM@fabric derived from the various IEM concentrations was measured, and the corresponding results for the hydrophobicity of the F-IEM@fabric are shown in Figure 2a. It could be found that, when only 2% IEM was utilized, the WCA of thew F-IEM@fabric could reach 148°, exhibiting quite a remarkable phenomenon compared with that of the pristine fabric (WCA~0°). While the mass concentration of IEM further increased from 2% to 8%, the WCA increased significantly from 148° to 162°. Afterwards, the WCA reached a plateau and remained at 162°, even when the concentration of IEM increased to 10%. The reason for this phenomenon may be that the IEM at a concentration of 8% completely reacted with the hydroxyl groups on the surface of the pristine fabric, and the grafted IEM on the surface of the fabric reached a saturated state. Even if the IEM concentration was further increased, the IEM on the surface would not increase. Therefore, when the IEM concentration climbed to 10%, the corresponding WCA remained at 162°, which did not further increase with the IEM concentration increasing. Considering the cost of raw materials and the corresponding WCA effect, the 8% concentration of IEM and equimolar amount of DFMA were selected as the best formula, and the corresponding F-IEM@fabric product was used for further characterization and testing.

In order to explore the grafting situation of the modified fabric under the optimal conditions, the FTIR spectra characterization of the pristine fabric, IEM, DFMA, and F-IEM@fabric are shown in Figure 2b. For the spectrum of IEM, it was clearly observed that an obvious absorption peak appeared at 2270 cm^−1^, which was attributed to the characteristic peak of –NCO [42,43]. However, no –NCO peak was observed at 2270 cm^−1^ in the spectrum of the F-IEM@fabric. This indicates that the –NCO group in the IEM reacted with the –OH in the fabric to form a urethane bond, causing the IEM to be chemically anchored onto the fibers’ surface. Meanwhile, compared with the pristine fabric, a new signal peak appeared at 1720 cm^−1^, which was derived from the C=O from IEM and DFMA [44,45]. In addition, in the spectra of IEM and DFMA, the characteristic peak at 1626 cm^−1^ was observed, which should be attributed to C=C [46], but this peak was not observed in the spectrum of the F-IEM@fabric, indicating that the C=C between IEM and DFMA had undergone a coupling reaction. Furthermore, compared with the stretching vibration peak of the C-F bond at the absorption band of 1210 cm^−1^ in DFMA [47], this peak was also clearly observed. All these verified that DFMA and IEM had successfully grafted onto the fabric’s surface under UV-light radiation.

To further evolute the fabric’s chemical composition before and after modification, XPS, as frequently employed method [48], was used to characterize the pristine fabric and F-IEM@fabric. Figure 2c demonstrates the elemental composition of the pristine fabric and F-IEM@fabric. The pristine fabric only showed the C1s peak (285.3 eV) and O1s peak (532.4 eV), which indicated that the pristine fabric was only composed of the elements C and O [49,50,51,52]. After being modified by IEM and DFMA, compared with the pristine fabric, the F-IEM@fabric had two new signal peaks, which were located at the F1s peak at 689 eV [53,54] and the N1s peak at 400 eV; this was caused by the appearance of N and F elements on the F-IEM@fabric. which were derived from IEM containing –NCO and DFMA containing the fluorine long chain, indicating that IEM and DFMA had been successfully introduced onto the fabric’s surface. Figure 2d,e show the high multiple C1s peaks of the pristine fabric and F-IEM@fabric. The high multiple C1s peak of the pristine fabric showed three binding energy peaks at 284.7 eV, 286.4 eV, and 287.7 eV, which correspond to the CHx [55], C–O, and C=O bonds. For the F-IEM@fabric, two new peaks at the binding energies of 285.4 eV and 293.3 eV occurred, which were derived from the C–N and C–F_3_ groups [54,56]. The above results prove the fact that IEM and DFMA had been successfully introduced to the fabric’s surface via a one-step modification strategy, which ultimately led to excellent superhydrophobic performance for the fabric.

### 3.2. Surface Morphology of the Superhydrophobic F-IEM@fabric

**Figure 3:**
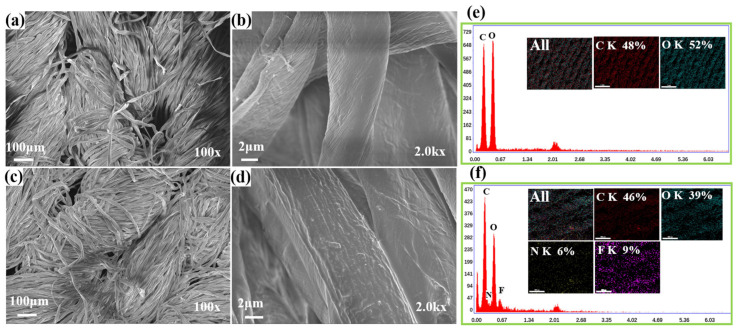
SEM images of (**a**,**b**) pristine and (**c**,**d**) F-IEM@fabric; EDS of (**e**) pristine fabric and (**f**) F-IEM@fabric.

Generally, the wettability of a fabric’s surface is closely related to its surface morphology [40,57]. The surface morphology of the pristine and modified samples was intuitively observed and analyzed by the SEM images, which are shown in Figure 3a–d. At low magnification, there was no obvious difference between the pristine fabric and F-IEM@fabric. As for the pristine fabric under the high-magnification SEM image (Figure 3b), its fiber’s surface was quite smooth, except for some natural wrinkles. After modification, the surface of the fibers showed completely different morphological characteristics, as seen in Figure 3d. It can be visually observed that the fibers’ surface was wrapped by some fish-scale-like protrusions, resulting in the appearance of a rough structure. These protrusions should be ascribed to the grafted IEM and DFMA molecules. In the fibers’ surface, there were large amounts of long chains, which should be randomly accumulated together. Due to the wrinkle effect [58], as well as the compatibility discrepancy between the fluoride group and IEM molecules, these long chains of grafted IEM and DFMA would form the fish-scale-like protrusions and rough structure on the fabric’s surface [59].

Furthermore, the element distribution and content variation before and after modification were characterized by EDS. As shown in Figure 3e,f, the C and O elements were dominant in the original fabric. After modification, two new elements appeared, which were attributed to the N and F elements that came from the isocyanate of IEM and DFMA, respectively. This indicated that the IEM and DFMA were successfully introduced to the fabric’s surface through the chemical grafting strategy, resulting in an increase in the hydrophobicity of the F-IEM@fabric. Normally, increasing the rough structure and decreasing the surface tension are vital to achieve superhydrophobicity [31,32], and both the surface micro-/nano-rough structure and surface chemical property are crucial in determining the hydrophobicity. The reason for the excellent superhydrophobicity of the F-IEM@fabric can be explained based on the rough structure and surface-modified molecules. On the one hand, as shown in the SEM images of the pristine (Figure 3a,b) and modified fabric (Figure 3c,d), the fibers in the pristine fabric were smooth accompanied by some inherent wrinkles, but those in the F-IEM@fabric were wrapped by some fish-scale-like protrusions, which should be the grafted IEM and DFMA. After the UV-light-induced coupling reaction completed, the coupled IEM and DFMA long chains would accumulate together and form the rough structure with the fish-scale-like protrusions, due to the wrinkle effect and compatibility discrepancy between the IEM and DFMA molecules [58]. On the other hand, the fluoride element has the lowest surface tension in nature, and there are twelve fluoride atoms in the DFMA molecule. A large amount of fluoride atoms were grafted and distributed on the fibers’ surface after the surface modification, which could be verified by the EDS results in Figure 3f, where the F element content was 9%; thus, the surface energy of the fiber greatly decreased compared to that before the modification. Therefore, the formed fish-scale-like rough structure, combined with the greatly decreased low surface energy, endowed the F-IEM@fabric with excellent superhydrophobic performance.

### 3.3. Surface Property of the Superhydrophobic F-IEM@fabric

**Figure 4:**
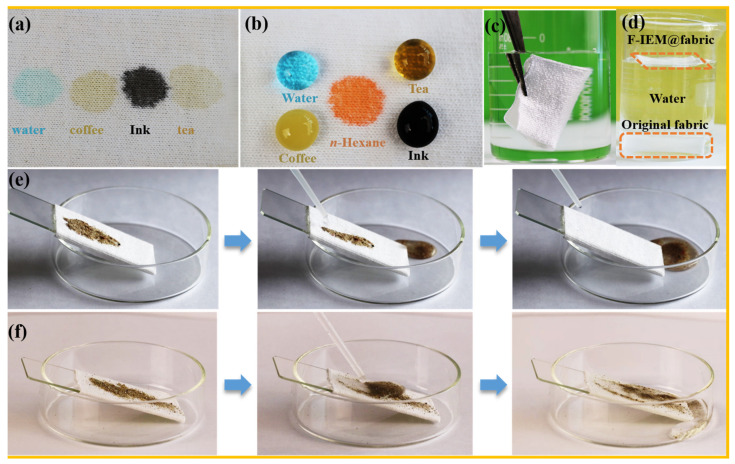
Image of liquid stains on (**a**) pristine fabric and (**b**) F-IEM@fabric; (**c**) the silver mirror phenomenon of F-IEM@fabric; (**d**) photos of putting pristine fabric and F-IEM@fabric into water. Self-cleaning test on the surface of (**e**) F-IEM@fabric and (**f**) pristine fabric.

The special surface property of selectively absorbing oil, but repelling water was fundamental for the F-IEM@fabric to separate the oil–water mixture. The surface property of the superhydrophobic F-IEM@fabric was evaluated by leaving various liquid droplets and monitoring their static behavior on the surface of the F-IEM@fabric, which are shown in Figure 4a,b. Water, coffee, ink, and tea were employed as water-soluble stains to assess the moisture resistance of the F-IEM@fabric. When these water-soluble stains approached the pristine fabric, it can be clearly observed that the droplets were instantly absorbed and gradually spread around the surface as a circle, which agrees with the superhydrophilic characteristic of WCA = 0°. In contrast, these droplets could not penetrate into the surface of the superhydrophobic F-IEM@fabric; they stood on the surface of the fabric in the shape of a ball and maintained the shape even after 1 h, which is in agreement with the superhydrophobic characteristic of WCA = 162°. For oily droplets of *n*-hexane, when they were dropped onto the F-IEM@fabric, the *n*-hexane droplets immediately absorbed and diffused on the fabric. The above phenomena revealed that the F-IEM@fabric possesses excellent resistance to water-soluble stains in air and prominent superhydrophobic/superoleophilic properties. Besides the superhydrophobic F-IEM@fabric exhibiting water-proof behavior in air, the F-IEM@fabric still had satisfying moisture resistance underneath water. When the superhydrophobic F-IEM@fabric was completely immersed into water, the silver mirror phenomenon was observed (Figure 4c), which should be attributed to the entrapped air cushion between the water and the modified fabric’s surface. Acting as a shielding, this air cushion can effectively inhibit water from penetrating into the fabric’s surface even if the fabric is fully immersed in water. When the pristine fabric and superhydrophobic F-IEM@fabric were put into water, respectively (Figure 4d), it can be clearly observed that the pristine fabric absorbed the water and gradually sunk to the bottom of the beaker under the action of gravity. On the contrary, even the superhydrophobic F-IEM@fabric was placed on the water surface for a long time, and it always floated due to its excellent superhydrophobicity. When F-IEM@fabric was taken out of the water, no water droplets were attached to the surface of the F-IEM@fabric, indicating that the F-IEM@fabric still had ideal moisture resistance in water. The moisture-proof property of superhydrophobic F-IEM@fabric is helpful to find application in self-cleaning fields. As shown in 4e, a layer of fine sand was spread on the surface of the superhydrophobic F-IEM@fabric; using a continuous stream of water to wash the fabric (see Video S1, Supplementary Materials), the falling water stream rolled off the surface while taking away the attached sand, resulting in a clean surface. In contrast, when the same operation was repeated on the surface of the pristine fabric (Figure 4f), the pristine fabric with superhydrophilic performance was quickly wetted by the water to form a water film, resulting in the sand adhering to the fabric’s surface tightly, which was difficult to wash away by the water stream; thus, stains remained on the surface of the fabric, showing a lack of the self-cleaning effect. These results imply that the superhydrophobic F-IEM@fabric is promising to be utilized in self-cleaning due to its special superhydrophobicity.

### 3.4. Breathable Property of Superhydrophobic F-IEM@fabric

**Figure 5:**
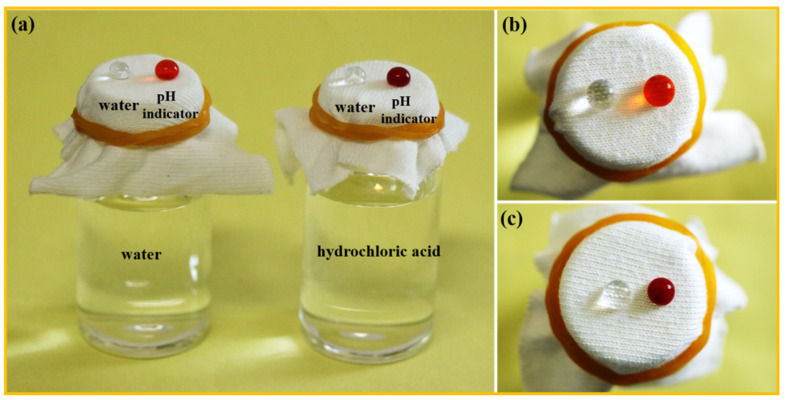
(**a**) Schematic diagram of the air permeability test. Top side view of the status of the water and pH indicator droplets in the case of water (**b**) and hydrochloric acid (**c**).

Besides the special superhydrophobicity, its porous structure forms the separation sites and access to achieve oil–water separation. After grafting the IEM and DFMA molecules, the anchored substances might block the pores of the fabric; thus, it is essential to evaluate the intrinsic breathability. Not only superhydrophobicity should be considered, but also the breathability of fabric should be taken into account together. Figure 5a shows the test of the waterproofness and breathability of the F-IEM@fabric. The superhydrophobic F-IEM@fabric was used to cover the mouth of a bottle containing water and a hydrochloric acid solution, respectively. In the case of the water, when the water and methyl-orange (pH indicator) droplets were placed onto the surface of the F-IEM@fabric, both the water and methyl-orange droplets stood on the fabric’s surface with a round shape. Even 10 min later, the water and methyl-orange droplets did not spread and penetrated the fabric from their top side view (Figure 5b), indicating the excellent water repellency of the F-IEM@fabric. In the case of the hydrochloric acid, a similar phenomenon was observed in which both the water and methyl-orange droplets still stood on the fabric’s surface with a round shape. It needs to be noted that the color of the pH indicator did not change in the case of the water, while in the case of the hydrochloric acid, the color of the pH indicator droplet gradually changed from orange to red, indicating that the methyl-orange droplet encountered an acidic atmosphere. This should be attributed to the volatilization of the hydrochloric acid; the volatile hydrogen chloride gas would pass through the fabric and meet the methyl-orange droplet, causing the methyl-orange droplet to be acidic and its color to gradually change from orange to red (Figure 5c). Based on the above phenomena, the superhydrophobic property was achieved after the facile one-step surface modification, and the breathable property was retained, which made the superhydrophobic F-IEM@fabric suitable to be utilized in oil–water separation.

### 3.5. Oil/Water Separation Performance of F-IEM@fabric

**Figure 6:**
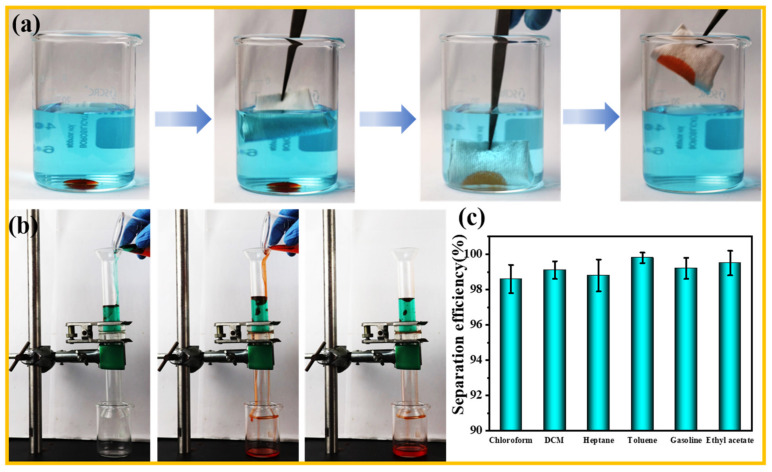
(**a**) The separation process of DCM–water mixture by F-IEM@fabric. (**b**) The continuous oil–water separation process of the F-IEM@fabric for DCM–water mixture. (**c**) The oil–water separation efficiency of the F-IEM@fabric for various organic solvents.

In view of the special wettability performance, the F-IEM@fabric is an ideal material to execute oil–water separation. As shown in Figure 6a, when the superhydrophobic F-IEM@fabric was immersed into the bottom of the DCM–water mixture, DCM was immediately absorbed by the F-IEM@fabric. Upon the completion of absorption, the fabric was taken out without residual oil droplets left in the water (see Video S2, Supplementary Materials), indicating the superhydrophobic F-IEM@fabric had a superior oil absorption ability and could instantly separate the DCM from the oil–water mixture. In practical applications, besides this intermittent separation, the continuous separation of the oil–water mixture is more commonly required in the case of large oil spills. Figure 6b shows the continuous separation operation of the F-IEM@fabric for the oil–water mixture. Taking a mixture of 20 mL de-ionized water and 20 mL DCM, where the water was dyed with anhydrous copper sulfate and the DCM was dyed with Sudan II, a simple set of equipment was set up where the superhydrophobic F-IEM@fabric was sandwiched between two glass tubes, as shown Figure 6b. The DCM–water mixture was poured slowly from the top into the glass tube, and the DCM sunk to the lower layer of the mixture due to its higher density than water. Benefiting from the superhydrophobic/superoleophilic properties of the F-IEM@fabric, the DCM was instantly absorbed by the F-IEM@fabric, and gradually, a layer of DCM formed on the fabric’s surface, leading to the increase of the contact area with the oil droplets. According to the theoretical intrusion pressure formula as follows [60,61]:

$$\Delta p=\frac{2\gamma }{R}=-\frac{L\gamma \cos\theta }{A}$$

where *γ* represents the oil liquid surface tension, R is the radius of the meniscus, and *L* and *A* are the circumference and area of the fiber pore, respectively. θ is the advancing angle of the oil droplets on the fabric. From this equation, it can be deduced that, when the F-IEM@fabric contacts oil, θ is less than 90°, cosθ is positive, and Δ*p* < 0; thus, the F-IEM@fabric cannot bear any pressure from the oil, and oil can easily penetrate into the fabric. In the conditions of water, θ is larger than 90°, cosθ is negative, and Δ*p* > 0, indicating the water can be supported. For the case of the DCM–water mixture, the DCM gradually penetrated into the pores of the fabric’s fibers and ultimately flow slowly into the beaker under the assistance of gravity, while the water was retained on the surface of the F-IEM@fabric. Finally, the DCM was successfully separated and collected (see Video S3, Supplementary Materials). For the case of the oil–water mixture, where the solvent density is less than water, an external negative atmosphere is required. The fabric could be placed on the top of the solvent phase, and the solvent could be absorbed by the fabric and transported to the beaker under the assistance of a vacuum pump. To further investigate the oil–water separation performance of the F-IEM@fabric, six organic solvents were selected to measure its oil–water separation efficiency, which is shown in Figure 6c. Among these six organic solvents, the highest separation efficiency was 99.8% for the toluene–water mixture, and the lowest separation efficiency was 98.6% for the chloroform–water mixture. All separation efficiencies were above 98%, indicating that the prepared superhydrophobic F-IEM@fabric had significant oil–water separation performance for most organic solvents.

### 3.6. Tolerance of the F-IEM@fabric in Harsh Environments

**Figure 7:**
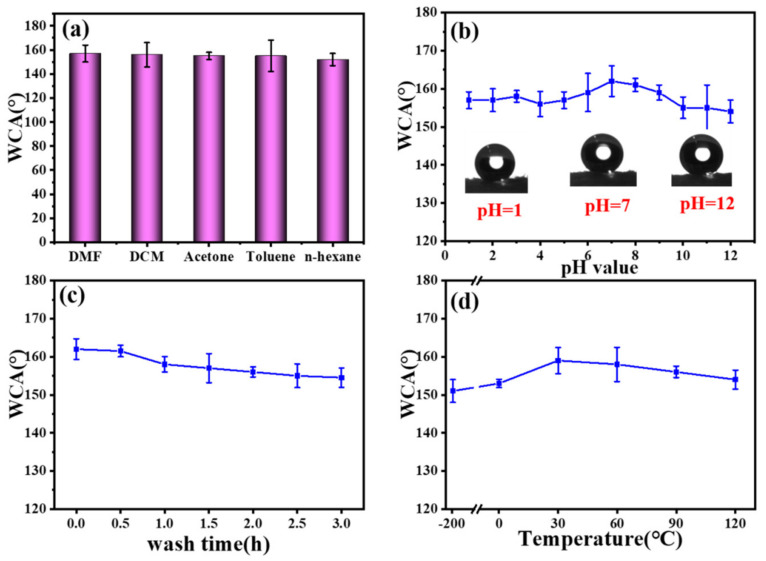
The WCA variation of the F-IEM@fabric after (**a**) immersion in various organic solvents for 72 h, (**b**) immersion in various solutions with various values of pH for 48 h, (**c**) laundry washing for 3 h, and (**d**) exposure to extreme high or low temperatures for 2 h.

In our daily lives, the practical application of superhydrophobic surfaces would probably encounter harsh environments, and most of them are vulnerable to mechanical damage or harsh conditions such as acids/alkalis, extremely high or low temperatures, etc., leading them to lose their superhydrophobicity. Therefore, it is essential to investigate the durability under harsh environmental conditions [62,63]. Herein, the stability of the F-IEM@fabric was evaluated by investigating the WCA changes of the F-IEM@fabric under extreme harsh conditions such as solvents, acids, alkalis, laundry washing, and high/low temperatures. After the F-IEM@fabric was soaked in various organic solvents for 72 h, the corresponding WCA changes are shown in Figure 7a. It can be observed that the maximum WCA 157° happened in DMF, and the minimum WCA was 155° in the ethanol solution. Although the resistance of the F-IEM@fabric in ethanol was weaker than the other organic solvents, it remained above 155°. In other words, the solvents had little effect on the superhydrophobicity of the F-IEM@fabric. Moreover, Figure 7b shows the corresponding WCA after immersing the F-IEM@fabric in a wide pH range solution (1–12) for 48 h. It can be seen that the WCA was the highest one when the pH (6–8) was approximately neutral, and this should be ascribed to the mild conditions of solutions with the acidic or alkali property increased, the pH decreased from 7 to 1, or the pH increased from 7 to 12; the WCA presented a slight decrease than the case of pH = 7, indicating the pH of the solution can exert some effect on the WCA of the fabric’s surface; this should be associated with the damage to the materials deriving from the IEM and DFMA molecules. Fortunately, the F-IEM@fabric can always maintain its superhydrophobic state. This demonstrates that the F-IEM@fabric has outstanding chemical stability even facing the attack of acidic and alkali solutions. The possible reason might be ascribed to the superior anticorrosive performance of the incorporated molecules whose chains contain the fluoride element and groups. Figure 7c shows the WCA variation of the F-IEM@fabric after the fabric was put into a laundry machine, which worked in the normal washing mode at the room temperature of about 25 °C, which is quite common for cleaning in our daily lives. With the washing time increased to 3 h, the WCA of the F-IEM@fabric decreased slightly from 162° to 155°. Additionally, the WCA variation of the F-IEM@fabric was also measured when the F-IEM@fabric was exposed to extreme high or low temperatures for 2 h, and the results are shown in Figure 7d. It can be clearly observed that the WCA had a maximum value when external temperature was around room temperature. As the temperature gradually increased to 120 °C or decreased to −196 °C, the WCA of the F-IEM@fabric had a certain degree of reduction, but still retained its superhydrophobicity, implying the outstanding resistance to extreme temperatures. All these results demonstrated that the prepared superhydrophobic F-IEM@fabric had outstanding tolerance to harsh conditions such as organic solvents, acidic or alkali solutions, laundry washing, and extreme temperatures.

### 3.7. Mechanical Durability of the F-IEM@fabric

**Figure 8:**
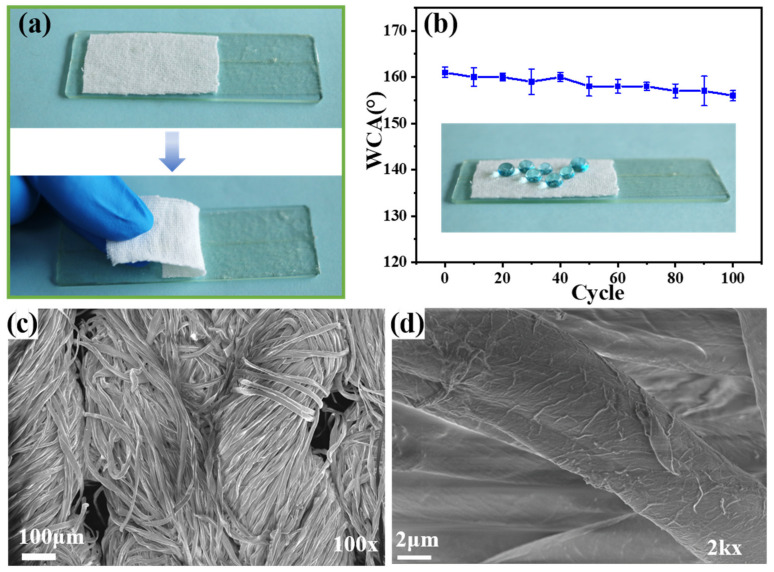
(**a**) The tape-peeling process of F-IEM@fabric, (**b**) the WCA variation of the F-IEM@fabric during 100 cycles of tape-peeling, and (**c**,**d**) SEM images of the F-IEM@fabric after 100 cycles of tape-peeling at low and high magnification.

In addition to harsh environmental conditions, fabrics commonly suffer from damage in daily applications and would lose their superhydrophobicity. Therefore, the mechanical durability of its superhydrophobicity is crucial in practical applications. Here, the durability of the superhydrophobic F-IEM@fabric against mechanical damage was evaluated by tape-peeling and abrasion tests. Figure 8a shows the tape-peeling process. First, the F-IEM@fabric was fixed on a glass plate by a double-sided tape, then the F-IEM@fabric was peeled off from the tape. Figure 8b presents the WCA variation of the F-IEM@fabric undergoing 100 cycles of tape-peeling. It can be clearly seen that the WCA had a slight downward trend with the cycles of tape-peeling increasing. After 100 cycles of tape-peeling, the F-IEM@fabric still maintained remarkable superhydrophobicity (WCA~156°). The reason for the decrease in the WCA may be related to the change of the surface morphology after the tape-peeling test, so the morphology of the F-IEM@fabric is explored in Figure 8c,d, which depict the SEM images of the F-IEM@fabric at low magnification and high magnification. Figure 8c shows that the overall appearance of the F-IEM@fabric was fluffy and neatly arranged with almost no visible alteration compared to the pristine fabric at low magnification. However, seen from the SEM image of a single fiber at high magnification in Figure 8d, there were numerous wrinkles and scaly substances sticking onto the fiber’s surface. This should be attributed to the fact that a small amount of coating layer on the fiber’s surface was scraped off by the sticky substance on the tape, accompanying the some of the sticky substance remaining after the tape-peeling test. Since the whole fiber still maintained an integral rough structure, the damage to the rough structure of its surface was almost negligible. This should be associated with the strong interactions between the fabric fibers and the grafting of IEM and DMFA, which were covalently bonded together. Therefore, the WCA of the F-IEM@fabric after 100 cycles of tape-peeling only exhibited a slight decrease, but always remained above 156°.

**Figure 9:**
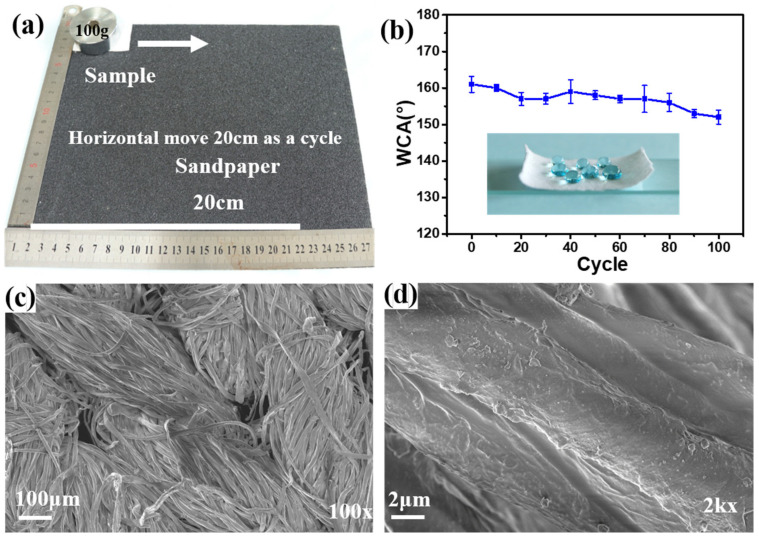
(**a**) The abrasion test process of F-IEM@fabric, (**b**) the WCA variation of F-IEM@fabric during 100 cycles of abrasion, and (**c**,**d**) SEM images of the F-IEM@fabric after 100 cycles of abrasion at low and high magnification.

Furthermore, for the durability of the F-IEM@fabric against abrasion, abrasion tests were also conducted. Figure 9a shows the abrasion test process for the F-IEM@fabric. First, the F-IEM@fabric was placed on sandpaper with a 300 mesh followed by a 100 g balancing weight on the fabric. The F-IEM@fabric was moved forward 20 cm at rate of 20 cm/min under external drag force. Figure 9b presents the corresponding WCA variation during 100 abrasion cycles. Overall, the WCA variation showed a deceasing trend, although the WCA fluctuated slightly. After 100 abrasion cycles, the WCA of the F-IEM@fabric declined to 153°. The reason for the decline should be ascribed to the external damage to the F-IEM@fabric’s surface by the abrasion. Similarly, Figure 9c,d show the surface morphology of the F-IEM@fabric after the 100-cycle abrasion test. Figure 9c shows for the overall morphology of the fibers in the F-IEM@fabric that no obvious changes were observed compared with before the abrasion, implying that the abrasion did not cause a huge visible damage to the F-IEM@fabric’s surface. However, it is not ignorable that there was some particulate matter scattered on the partial surface (Figure 9d), which should be ascribed to the crushing of heavy objects during abrasion. However, the overall rough structure of the fibers’ surface did not suffer great damage, and this should be ascribed to the strong covalent interactions between the fabric fibers and the grafted IEM and DMFA substances. It is reasonable that the WCA of the F-IEM@fabric did not significantly decline, and the F-IEM@fabric still exhibited remarkable superhydrophobicity.

### 3.8. Method Comparison with Recent Similar Studies

Table 2 compares this method with similar studies. Compared with the recent studies, this method in this article exhibited superiority in the following aspects. On the one hand, most of the reported methods require nanoparticles to construct a rough structure to reach superhydrophobic performance. Note that the SiO_2_ nanoparticles are popular and mostly used due to their white appearance and low cost; therefore, the resultant superhydrophobic fabric is white. Except the SiO_2_ nanoparticles, other nanoparticles are colorful such as the dark magnetite (Fe_3_O_4_) and polydopamine (PDA); thus, this would bring some negative effects regarding the appearance of the fabric. Additionally, these nanoparticles might block the pores of the fabric, and they easily detach from the fabric’s surface due to the weak interaction between them and the fabric fibers; thus, it is in imperative to avoid using these nanoparticles during the preparation process. In this article, no nanoparticles were required, and the IEM-DFMA molecules were grafted onto fabric; thus, the fabric not only can retain its intrinsic color, but also can retain the durable superhydrophobicity due to the strong covalent interaction with the fabric. On the other hand, although there are some methods in which no nanoparticles are involved, these methods require at least two steps and/or a long time to prepare the superhydrophobic fabric, which is very time consuming and inefficient. Remarkably, this article provides a facile, one-step high-efficiency method to prepare the superhydrophobic fabric under the aid of UV light.

## 4. Conclusions

In summary, a facile and efficient strategy was developed to prepare fabric with durable superhydrophobic properties. The pre-treated hydroxylated fabric was completely immersed into the solutions of IEM, DFMA, and photo-initiator, and the IEM containing isocyanate reacted with the hydroxyl groups on the fabric’s surface; meanwhile, the IEM and DFMA terminal double bonds were coupled under the assistance of ultraviolet light. The synergistic effect of the formed rough structure and low-surface-energy substance contributed to the significant superhydrophobicity of the F-IEM@fabric. Owing to its outstanding waterproof property, the superhydrophobic F-IEM@fabric could be used in the field of self-cleaning. When the superhydrophobic F-IEM@fabric was used for oil–water separation, it exhibited high separation efficiency over 98%. More importantly, the F-IEM@fabric exhibited excellent stability when exposed to harsh conditions such as organic solvents, acidic or alkali solutions, laundry washing, and extreme temperatures, as well as exhibiting durable superhydrophobicity against damage such as tape-peeling and abrasion tests. This was attributed to the stable covalent interactions formed among the fabric, IEM, and DFMA. Hence, this work provides a simple and efficient strategy for preparing a superhydrophobic fabric with excellent air permeability and stability, which is a promising candidate material in the application of oil–water separation.

## Figures and Tables

**Table 1:** Experimental formula for the preparation of F-IEM@fabric.

IEM Mass Concentration (%)	IEM (mol)	DFMA (mol)	DCM (g)	HMPF (g)
2%	0.005	0.005	40	0.2
4%	0.01	0.01	40	0.2
6%	0.015	0.015	40	0.2
8%	0.02	0.02	40	0.2
10%	0.025	0.025	40	0.2

**Table 2:** Comparison of the reported methods of superhydrophobic fabric.

Samples	Nanoparticles	Fabric Appearance	Operating Steps	Time (h)	Ref.
P-D-Fabric	SiO_2_	milky white	3	9	[64]
PEI/TMSPA/SiO_2_/DTMS cotton fabric	SiO_2_	pure white	2	20	[65]
APTES/IPDI/SiO_2_ fabric	SiO_2_	pure white	3	52	[66]
MCFs	TiO_2_	pure white	4	43.5	[67]
DSR-CZPP	ZnO	yellow	5	82	[68]
PA-Ploymer-Al_2_O_3_-Fabric	Al_2_O_3_	light yellow	3	30	[69]
Superhydrophobic Fabrics	Co_0.8_Mg_0.2_Fe_2_O_4_	brownish black	3	68	[70]
TSP-PET fabric	TiO_2_(sol)	light yellow	3	30	[71]
Fabric-SMP-S-SiO_2_@Fe_3_O_4_	MPS-SiO_2_@Fe_3_O_4_	brown	2	6.5	[40]
Mn@TiO_2_ membrane	Mn@TiO_2_	brown	3	17	[72]
FAS/SH-F-POSS fabric	POSS	pure white	3	11	[73]
Fe/PDA/ODA cotton fabric	PDA	black	2	5.5	[74]
PMT@fabric	○	light yellow	3	6	[75]
WSiPU-treated fabrics	○	milky white	2	3.5	[76]
F-IEM@fabric	○	white	1	1	This work

○ means no nanoparticles during the preparation.

## Data Availability

The data presented in this study are available on request from the corresponding author.

## Supplementary Materials

The following supporting information can be downloaded at: https://www.mdpi.com/article/10.3390/polym15112505/s1, Video S1: The self-cleaning process of superhydrophobic F-IEM@fabric surface; Video S2: The absorbing and collecting process of DCM from DCM–water mixture using superhydrophobic F-IEM@fabric; Video S3: The separation process of DCM–water mixture using superhydrophobic F-IEM@fabric.
